# Visual training after central retinal loss limits structural white matter degradation: an MRI study

**DOI:** 10.1186/s12993-024-00239-w

**Published:** 2024-05-24

**Authors:** Anna Kozak, Marco Ninghetto, Michał Wieteska, Michał Fiedorowicz, Marlena Wełniak-Kamińska, Bartosz Kossowski, Ulf T. Eysel, Lutgarde Arckens, Kalina Burnat

**Affiliations:** 1 Laboratory of Brain Imaging, Institute of Experimental Biology, Polish Academy of Sciences, Warsaw, Poland; 2 Faculty of Electronics and Information Technology, Warsaw University of Technology, Warsaw, Poland; 3 Small Animal Magnetic Resonance Imaging Laboratory, Mossakowski Medical Research Institute, Polish Academy of Sciences, Warsaw, Poland; 4 Department of Neurophysiology, Ruhr University, Bochum, Germany; 5 Laboratory of Neuroplasticity and Neuroproteomics, Department of Biology, KU Leuven, Louvain, Belgium; 6 KU Leuven Brain Institute, KU Leuven, Louvain, Belgium

**Keywords:** Retinal lesion, Visual training, Visual system, White matter structure, V5/PMLS, dLGN, Caudate nucleus, Hippocampus, FA, Fixel based analysis

## Abstract

**Background:**

Macular degeneration of the eye is a common cause of blindness and affects 8% of the worldwide human population. In adult cats with bilateral lesions of the central retina, we explored the possibility that motion perception training can limit the associated degradation of the visual system. We evaluated how visual training affects behavioral performance and white matter structure. Recently, we proposed (Kozak et al. in Transl Vis Sci Technol 10:9, 2021) a new motion-acuity test for low vision patients, enabling full visual field functional assessment through simultaneous perception of shape and motion. Here, we integrated this test as the last step of a 10-week motion-perception training.

**Results:**

Cats were divided into three groups: retinal-lesioned only and two trained groups, retinal-lesioned trained and control trained. The behavioral data revealed that trained cats with retinal lesions were superior in motion tasks, even when the difficulty relied only on acuity. 7 T-MRI scanning was done before and after lesioning at 5 different timepoints, followed by Fixel-Based and Fractional Anisotropy Analysis. In cats with retinal lesions, training resulted in a more localized and reduced percentage decrease in Fixel-Based Analysis metrics in the dLGN, caudate nucleus and hippocampus compared to untrained cats. In motion-sensitive area V5/PMLS, the significant decreases in fiber density were equally strong in retinal-lesioned untrained and trained cats, up to 40% in both groups. The only cortical area with Fractional Anisotropy values not affected by central retinal loss was area V5/PMLS. In other visual ROIs, the Fractional Anisotropy values increased over time in the untrained retinal lesioned group, whereas they decreased in the retinal lesioned trained group and remained at a similar level as in trained controls.

**Conclusions:**

Overall, our MRI results showed a stabilizing effect of motion training applied soon after central retinal loss induction on white matter structure. We propose that introducing early motion-acuity training for low vision patients, aimed at the intact and active retinal peripheries, may facilitate brain plasticity processes toward better vision.

## Background

It is well established that the adult mammalian brain has less capacity for structural and functional plasticity upon injury than a young brain. In past decades, many studies carried out on animal models aimed at achieving the feat of giving the postnatal plastic state back to the adult brain (e.g., [11, 17, 64]). Among different approaches, experimental induction of central retinal lesions to model retinal degradation similar to macular degeneration (MD), allows us to trace associated molecular, cellular and circuit dynamics occurring in an adult mammalian brain upon acquired sensory loss. Central retinal photoreceptor degeneration, as in MD, affects 8% of the worldwide human population [68]. It permanently removes central visual field processing while leaving the visual input from the periphery intact. Hence, we asked whether the ensuing reorganization of the peripheral visual field representations can be stimulated by visual training, leading to better functional compensation for the loss caused by central retinal damage in the animal model of MD. Previous neurophysiological and neuroanatomical (e.g., [23, 25, 26, 33, 35, 36]) as well as molecular investigations [2, 9, 31, 32, 49, 52, 62] of the mechanisms of topographical reorganizations following sudden central vision loss due to retinal damage have already revealed distinct neuroplasticity mechanisms on early levels of the adult visual system of cats, monkeys, and mice. However, only a few studies have explored the plasticity response to central retinal damage beyond the primary visual cortex (V1, cat: [3] and [15], monkey: [37]). Outside V1, we showed that for most of the higher order visual areas, the retinotopic lesion projection zone typically displayed an initial depression in expression of the molecular activity marker *zif268*, mirroring the sudden loss of visual input, followed by partial recovery with increasing postlesion time, reflecting cortical neuronal reactivation in cats [15]. Only motion-sensitive area V5/PMLS, which was not directly affected by the central lesions, showed no initial decrease in *zif268*. However, there was a significant activity increase at 12 weeks post-retinal lesion. In this animal model of MD, we thus showed a distinct form of neuroplasticity in the dorsal stream within a few months after the induction of central vision loss [15]. Similarly, behavioral tests of motion perception found no impairment but instead even better sensitivity to higher random dot stimulus velocities.

In the present study, we explored the possibility that motion perception training can limit the functional degradation of white matter fibers expected to occur after central retinal lesions in this cat model of MD. We used random dot kinematograms for motion-acuity training of the peripheral and central visual field simultaneously to monitor longitudinal changes in behavioral performance, relying on a task set that was previously developed as a new motion acuity test for patients with photoreceptor degeneration [38, 45]. To track corresponding structural changes in white matter, we performed a longitudinal, whole brain fixel based analysis (FBA) and Fractional Anisotropy Analysis (FA). FBA is a precise model of white matter analysis that considers the multiple directions of fibers within each voxel used for the identification of white matter changes in neurodegenerative disorders [29, 43]. As a measure of integrity of white matter structure the temporal changes in Fractional Anisotropy levels (FA) were assessed by region of interest (ROI) based analysis in visual areas 17, 18, 19, 21a, and posteromedial lateral suprasylvian area (PMLS), the homologues of primate areas V1, V2, V3, V4, and V5, respectively as described in our previous research on the cat MD model [15].

## Results

**Fig. 1:**
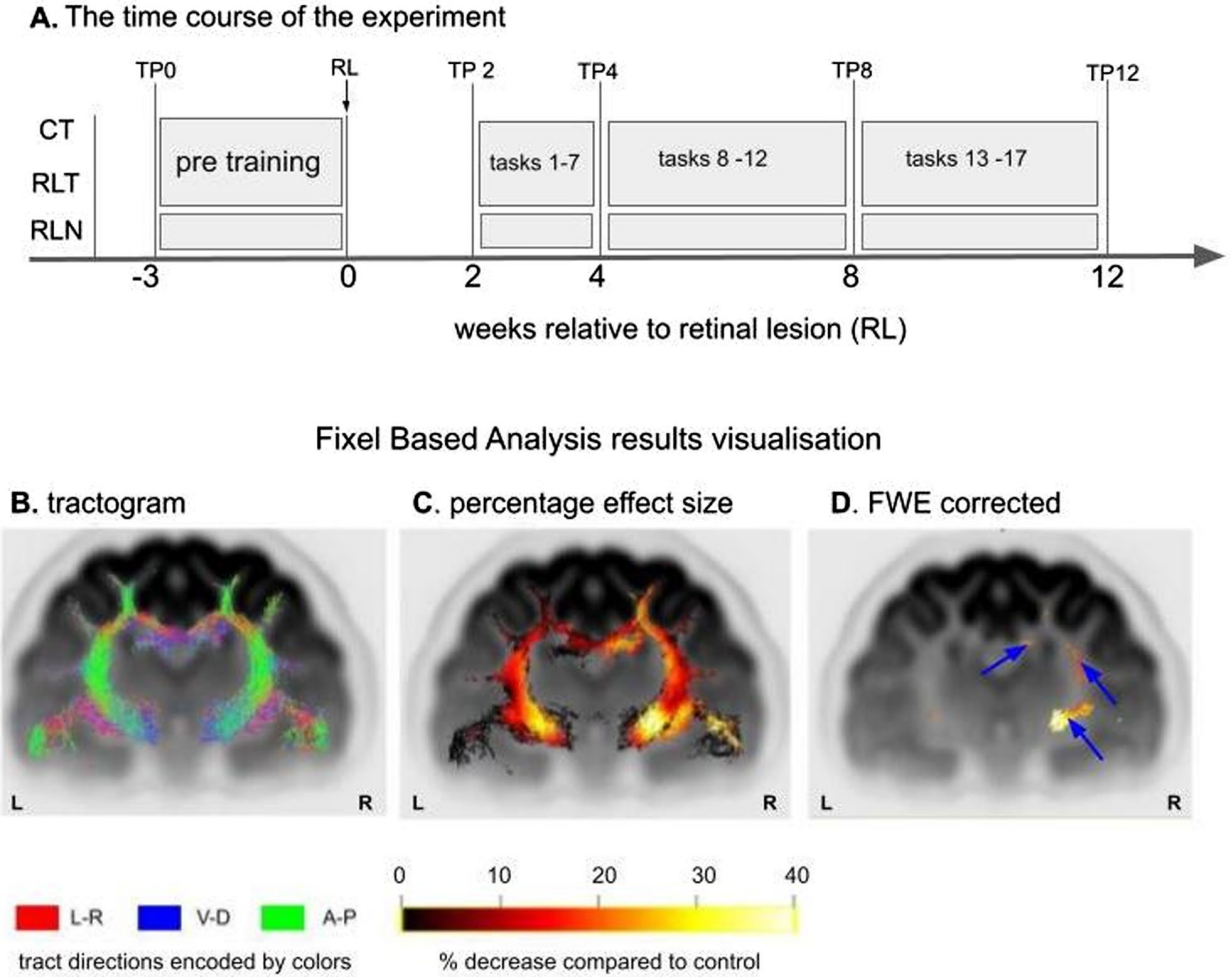
Experimental design and visualization of the FBA procedure. **A** Time course of the experiment: control trained (CT), retinal-lesioned trained (RLT), and retinal-lesioned naive (RLN) cats. MRI scanning sessions were performed at five time points (TP). TP0—the first MRI scanning was performed 3 weeks before the induction of retinal lesions (RL, indicated by an arrow, week 0). Next MRI scannings were performed after induction of RL at two weeks, TP2; four weeks, TP4; eight weeks, TP8; and twelve weeks, TP12. Consecutive motion tasks were performed between MRI scanning sessions, starting from postlesion weeks 2–4 (tasks 1–7), 4–8 (tasks 8–12) and 8–12 (tasks 13–18). Tasks are summarized in Table 1. RL induction was preceded by 3 weeks of pretraining for the control trained and retinal lesioned trained groups. **B**–**D** Steps leading to visualization of FBA results shown on an example coronal section. **B** Whole brain tractogram, with fiber bundle directions coded by colors: red, left–right; blue, ventral-dorsal; green, anterior–posterior. **C** The percentage effect compared to the control is shown by a color gradient from black (0%) to white (40%). **D** Cropped tractogram based on FWE = 0.05. Blue arrows denote the significant percentage decrease compared to the control

**Fig. 2:**
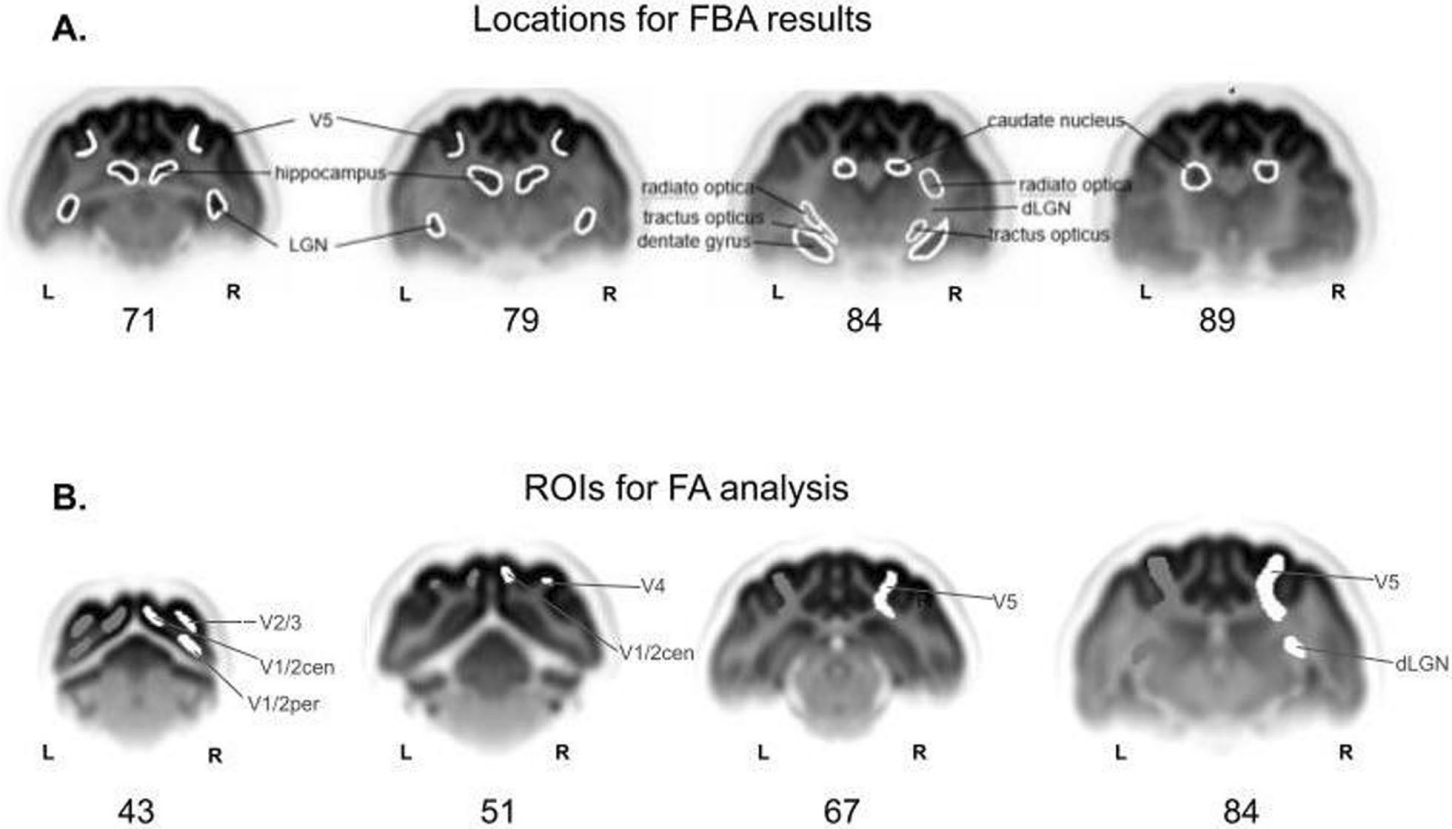
Delineation of areas for white matter structure analysis. **A** Areas in which the percentage decrease of the fixel based analysis metrics was revealed are drawn on the diffusion weighted image population template, based on the “Stereotaxic Atlas of the Cat Brain” (Snider, Ray S., Niemer, William T., and detailed description in [15]). **B** ROIs used for FA data analysis, drawn on the diffusion-weighted image population template in FSLeyes software, based on the same atlases mentioned above. ROIs were drawn for the left and right hemispheres. V1/2cen—inputs to the central representation of V1 and V2; V1/2per—peripheral representation. The numbers indicate the slice order in the population template shared for FA and FBA analysis, from posterior to anterior

7T-MRI scanning was performed at 5 different timepoints before and after lesioning the central retinas of adult cats. Figures 1 and 2 summarize the overall experimental setup for all cats, including the timing of lesion induction, visual training, MRI sessions and data analysis. All MRI sessions always involved cats from the three experimental conditions, control trained (CT), retinal-lesioned trained (RLT), retinal-lesioned naive (untrained; RLN). The first control MRI scannings were performed when the animals were 8 months old, at time point 0 (TP0, Fig. 1A). Then, all trained cats were familiarized with the automatic training apparatus and pretrained, as described in [70]. A second MRI scanning session took place after a two-week recovery period for retinal lesion cats (TP2, Fig. 1A and Methods). This time point was chosen as the first experimental condition because previous investigations have shown a clear functional [25] and maximal molecular response to the retinal lesion 2 weeks after lesioning [3]. At TP2 visual training began for the control and retinal-lesioned trained cats and more MRI scanning sessions took place 4, 8 and 12 weeks after retinal lesion induction (TP4, 8, 12, Fig. 1A).

### Fixel based whole-brain analysis of the percentage effect

**Fig. 3:**
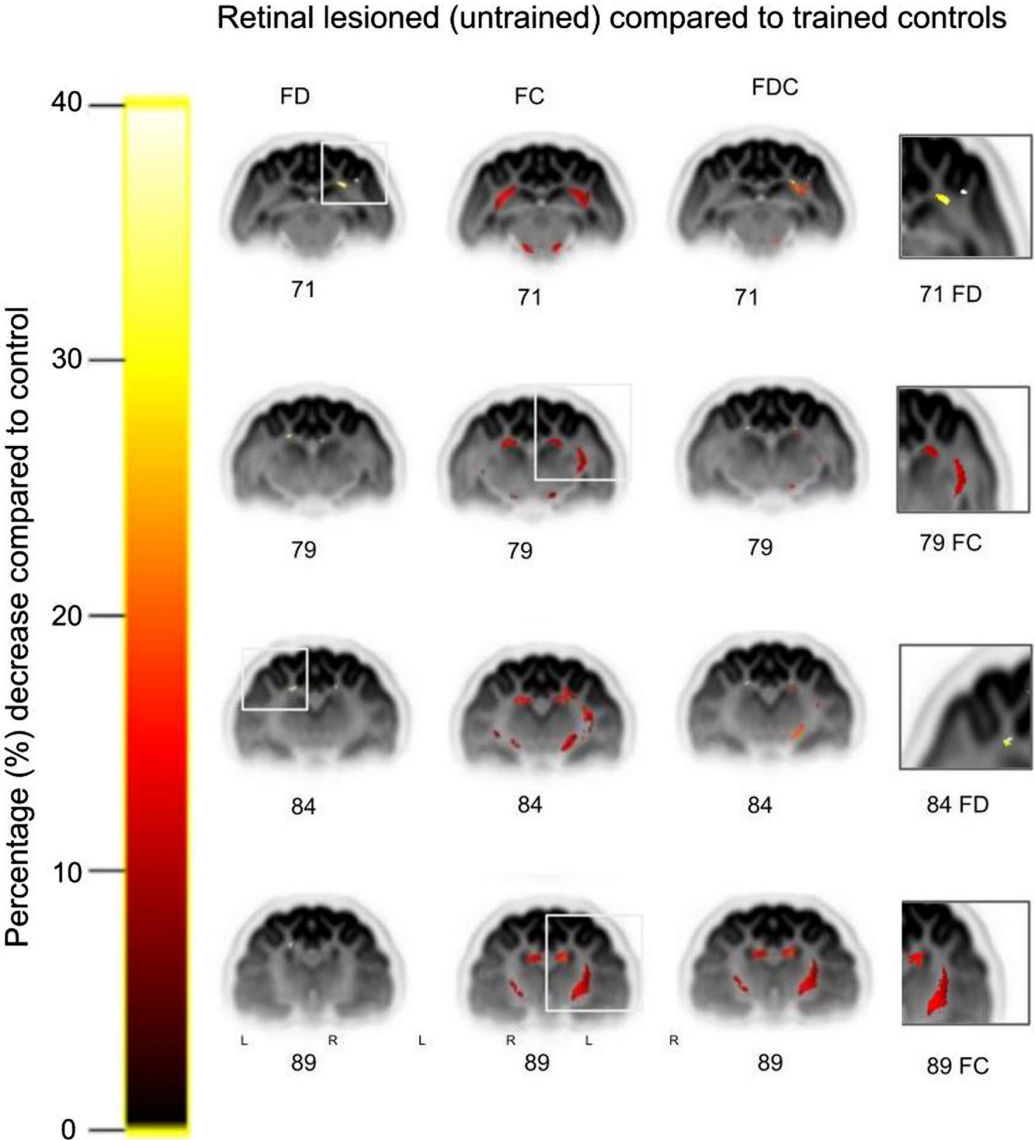
Comparison of retinal-lesioned naive versus control trained cats for fiber tract-specific significant percentage decreases. Significant streamlines are displayed across axial slices of the population template-based tractogram, in which streamlines were cropped to significant fixels only (FWE-corrected *p* value < 0.05) to visualize the regions implicated in the observed changes. Columns from left to right: fiber density (FD), fiber cross section (**FC**), fiber density and cross section (FDC) and magnification. White rectangles denote regions that are magnified and shown in the right column. Significant results were found for V5/PMLS (71 FD), dLGN and hippocampus (79 FC), hippocampus (84 FD) and dLGN and caudate nucleus 89 (FC). Note that the FD results presented in A. show a unilateral strong, up to 40% decrease in the V5/PMLS (slice 71)

**Fig. 4:**
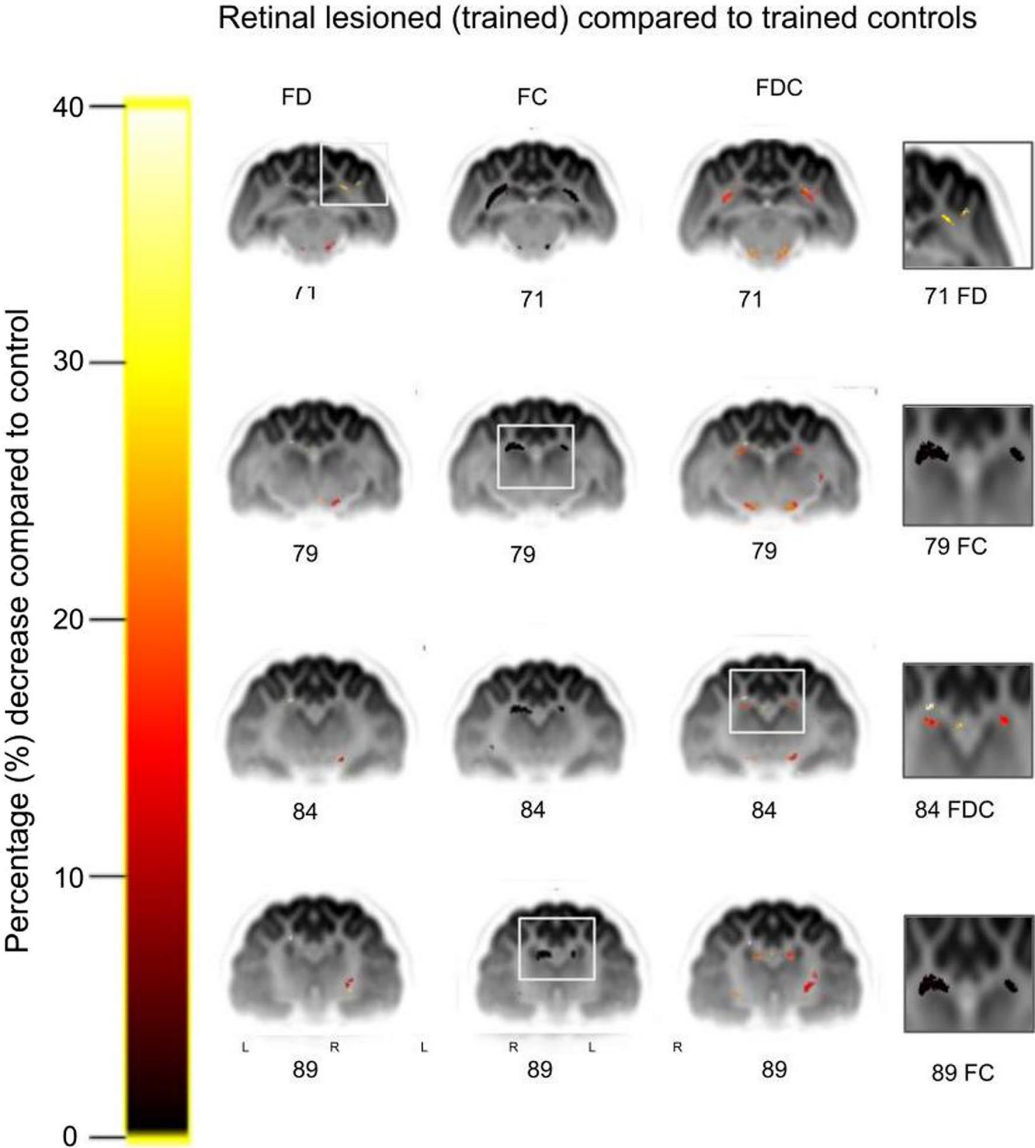
Comparison of retinal-lesioned trained versus control trained cats for fiber tract-specific significant percentage decreases. Significant streamlines are displayed across axial slices of the population template-based tractogram, in which streamlines were cropped to significant fixels only (FWE-corrected *P* value < 0.05). Columns from left to right: fiber density (FD), fiber cross section (FC), fiber density and cross section (FDC) and magnification. White rectangles denote regions that are magnified and shown in the right column. Significant results were found for the V5/PMLS (71 FD), hippocampus (79 FC), caudate nucleus (84 FDC) and caudate nucleus 89 (FC). Of note, the FD results for V5/PMLS of retinal-lesioned trained cats (slice 71) are similar to those for the retinal-lesioned naive group, as presented in Fig. 3 (slice 71)

In Figs. 3 and 4, we show the white matter streamlines pseudocolored for a significant percentage decrease in FBA metrics between conditions: microstructural fiber density (FD), macrostructural fiber bundle cross-section (FC), and the combination of FD and FC (FDC) compared to the control trained group (100%).

The retinal-lesioned naive group exhibited a decrease: in the FD metric—unilateral, focal decrease up to 40% in V5/PMLS and left hippocampus; in the FC metric—bilateral, widespread decrease up to 15% in the dLGN, caudate nucleus and dorsal hippocampus; in the FDC metrics—unilateral, focal, up to 20% decrease in the right dLGN and bilateral, widespread in dorsal hippocampus and caudate nucleus (Fig. 3). The retinal-lesioned trained group exhibited a decrease: in the FD metric—a unilateral focal decrease up to 40% in the right V5/PMLS; in the FC metric—bilateral widespread decrease up to 5% in the dLGN, caudate nucleus and dorsal hippocampus; and in the FDC metrics—bilateral widespread decrease up to 20% in the dLGN, dorsal hippocampus and caudate nucleus (Fig. 4).

Given the relatively large voxel size of the diffusion images (3 × 3 mm) and that 90% of the white matter voxels in the brain contain multiple fiber orientations [54], with FBA modeling of single fiber bundles, we were not able to detect any significant temporal changes in the white matter structure induced by lesion and/or training.

### Fractional anisotropy ROI analysis

**Fig. 5:**
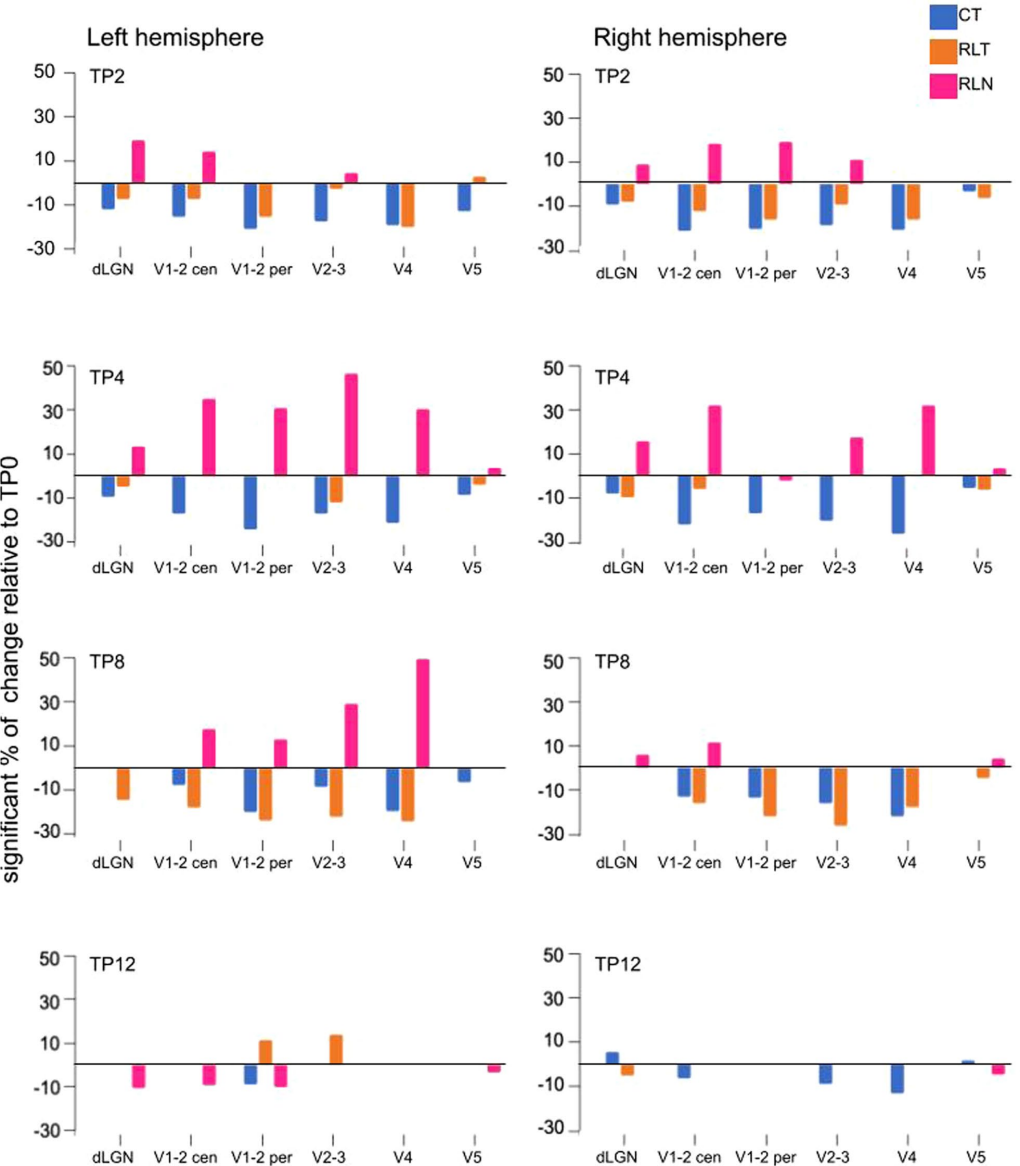
Representation of the significant FA changes during postlesion recovery relative to prelesion levels (TP0). White matter subcortical representation of the different ROIs in visual feedforward input order: dLGN, dorsal lateral geniculate nucleus; V1-2 cen, central region of V1 (area 17) and V2 (area 18); V1-2 per, peripheral region of V1 and V2; V2-3 (areas 18 and 19), V4 (area 21a) and V5 (area PMLS). The color of the bars represents the different groups of cats: blue, control trained; orange, retinal lesion trained; magenta, retinal lesion naive. The percentage of change was calculated from significantly different means with full factorial GLM analysis with the Bonferroni's post hoc test

The temporal changes in white matter structure were assessed by ROI-based analysis of FA. In Figure 5, we show, separately for each hemisphere and group of cats, the significant percentage of change in the FA values at TP2-12 compared to the FA level at TP0. In general, at TP2, 3 weeks of visual pretraining in the control trained group was accompanied by a significant decrease in the FA values in all analyzed ROIs in both hemispheres (Figure 5, blue bars), which stabilized at TP12, mainly in the left ROIs. In contrast, in the retinal-lesioned naive group, the induction of retinal lesions led to an increase in FA (Figure 5, magenta bars). The ROIs located in the right hemisphere reacted faster to the induction of the retinal lesions and reached lower FA levels again more quickly, similar to those measured at TP0 (3 weeks before lesioning). At TP2, that is, 2 weeks after lesion induction, areas V4 and V5/PMLS of retinal-lesioned naive cats did not show such a significant increase in FA in either hemisphere. The only detected increased FA levels were left ROIs, V1-2 peripheral and V2-3 at TP12 (Figure 5). In the retinal-lesioned trained group, at TP2, the FA values decreased, similar to control trained cats, normalizing at TP4, decreasing again at TP8, and finally normalizing again at TP12 (Figure 5, orange bars).

### Outstanding behavioral performance of the retinal-lesioned trained cats

**Fig. 6:**
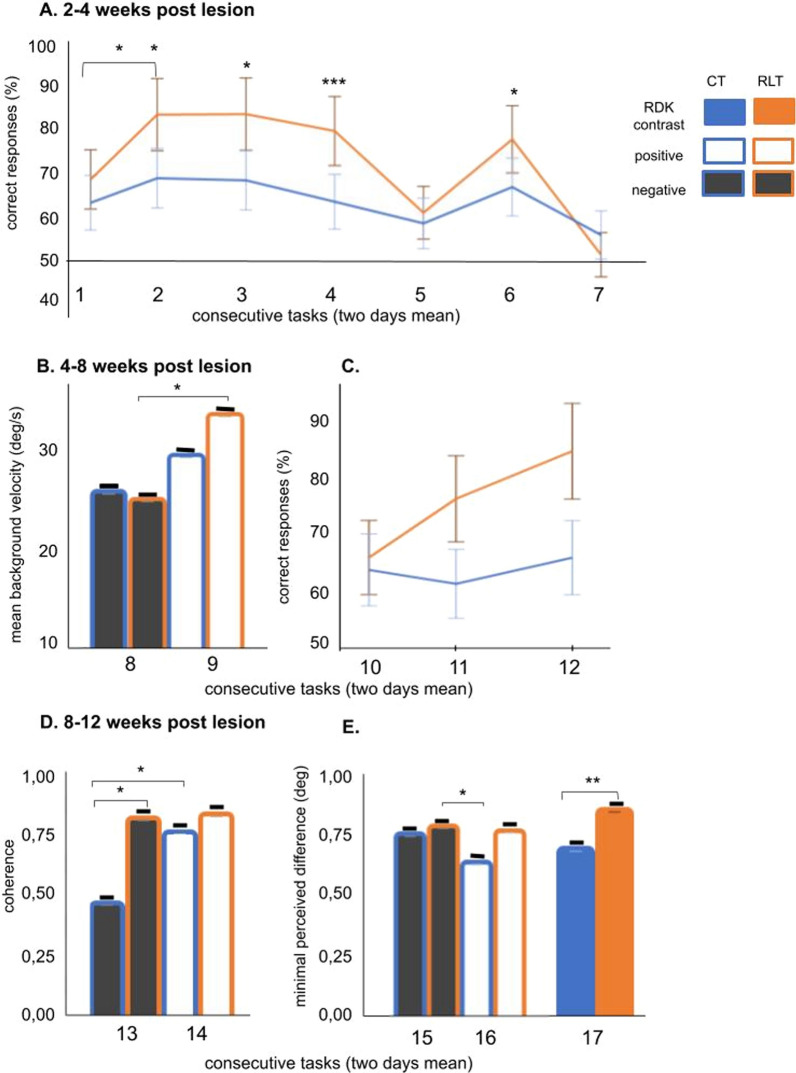
Superior visual performance of trained cats with retinal lesions. 2–4 weeks postlesion** A**. Contrast difference between tasks. Background RDK is introduced, from stationary (task 3) to motion at high velocity (tasks 5 and 7). 4–8 weeks postlesion (**B**, **C**). **B** The threshold level for background velocity. The task was significantly easier in positive contrast than in negative contrast in the retinal-lesioned trained group. **C** The direction of the background RDK is changed to horizontal**.** The first task (task 10) was as difficult for both groups. Next, retinal-lesioned trained cats performed better than control-trained cats when velocity became slower (tasks 11 and 12). 8–12 weeks postlesion (**D**, **E**). **D** The mean threshold for background RDK coherence. **E** Acuity tasks based on the shape difference between circle and ellipse. Retinal-lesioned trained cats performed significantly better than control trained cats in acuity discrimination of the plain gray shapes. (**A**) and (**C**) mean percentage of correct responses with standard deviation. **B** The threshold level for background velocity. **D** The mean threshold for background RDK coherence. **E** Minimal perceived difference. Significant differences are indicated by * p ≤ 0.05, ** p ≤ 0.01 and *** p ≤ 0.001, Tukey’s post hoc test. Colors of the bars denote stimulus contrast: dark gray, negative contrast; white, positive contrast. Numbers on the X axes denote the tasks, which are depicted in detail in Table 1

After initial pretraining, all cats were familiarized with the simple discrimination task, where S+/S- differed in shape (circle/ellipse) and direction of dot motion (downward/upward). Table 1 graphically summarizes the different perceptual tasks used for training: 2–4 weeks, 4–8 weeks and 8–12 weeks postlesion. For control trained animals, all perceptual tasks introduced during the 1st month of training were challenging, as reflected by the stable low performance (Fig. 6A, C, blue line). In contrast, retinal lesion-trained cats benefited from switching the contrast of S+/- RDKs from positive to negative (Fig. 6A, orange line, tasks 1 to 2). They continued to perform significantly better than control trained cats when background RDK was introduced (Fig. 6A, tasks 3 and 4). Only fast velocity of the background RDK significantly lowered retinal lesioned trained performance to the control trained level (Fig. 6A, task 6, 7).

**Table 1:**
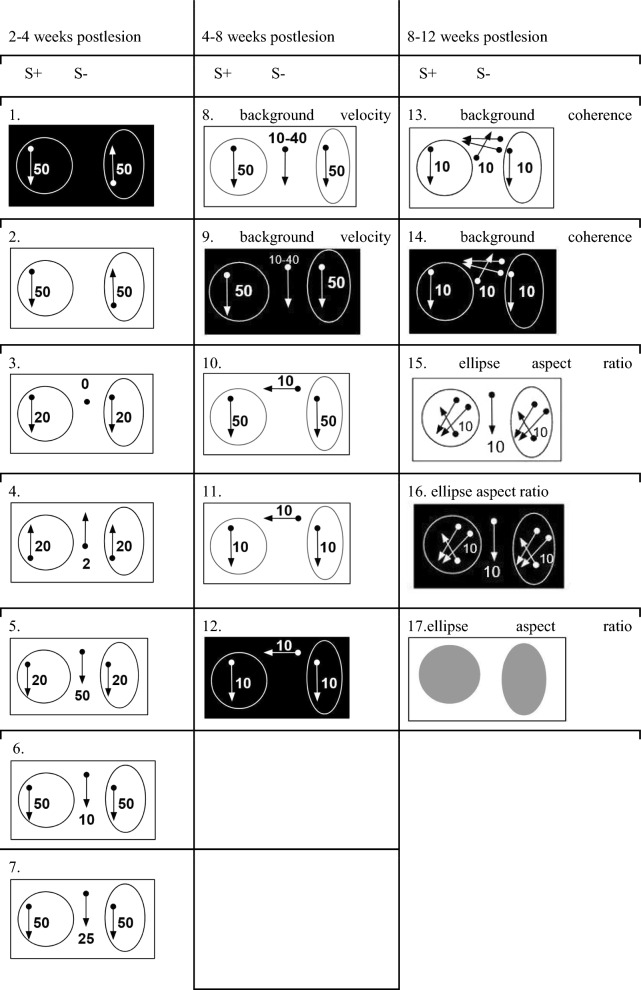
Schematic representation of the stimuli in the order of training

Each column shows stimuli that were assigned for analysis related to white matter measurements at timepoints TP4, 8, and 12. The direction of arrows denotes the direction of the moving dots. The numbers within the stimuli denote the velocity of the dots, given in deg/s. Constant stimuli procedure for tasks 1–7 and 10–12 and adaptive staircase procedure for tasks 8–9 and 13–17

### 4–8 weeks postlesion time

Using a staircase method adaptive to the animal performance, we first checked the background velocity thresholds for negative and positive contrasts separately, since we knew from the previous tasks that the velocity manipulations significantly affected the performance of the retinal lesioned trained group. For control trained cats, the thresholds for both contrasts were the same, and for retinal-lesioned trained cats, the positive contrast RDK was significantly easier and had a higher velocity threshold than in negative contrast (Fig. 6B).

Next, we wanted to verify the effect of the perpendicular direction difference between S+/- and the background. Therefore, we changed the motion direction of background dots from vertical (downward) to horizontal. Additionally, to remove velocity difficulty, the background velocity was decreased to 10 deg/s. This task was equally difficult for both groups (Fig. 6C). However, for retinal-lesioned trained cats, decreasing velocity in S+/- to 10 deg/s resulted in significant improvement of performance for both contrasts compared to control trained cats (Fig. 6C).

### 8–12 weeks postlesion time

In tasks 13 and 14, we measured the coherence of background RDKs at a velocity of 10 deg/s, which is optimal for retinal-lesioned trained cats, at the threshold level (tasks 13 and 14; Fig. 6D). Control trained cats achieved significantly lower thresholds in the negative contrast task compared to the retinal lesioned trained group and compared to their thresholds in the positive contrast (Fig. 6D).

In the last three tasks, we measured the acuity based on the shape difference between the circle and ellipse, in which the ellipse became more similar in shape to the circle (Fig. 6E). Motion acuity measurement was performed in the first two tasks (15, 16), the gradient between shapes was based on the coherence difference, and we tested again for the two contrasts. Finally, we removed the motion, leaving only the plain stationary gray shapes (task 17). In the stationary acuity task, the retinal-lesioned trained cats performed significantly better than the control trained cats (Fig. 6E, task 17).

## Discussion

In our animal model of central vision loss, we showed a stimulating effect of training applied in the early phase after lesion induction, which was demonstrated by the superior behavioral performance of retinal-lesioned cats compared to control subjects. The positive effect of training was corroborated by the white matter structure analysis. In retinal-lesioned cats, training resulted in a more localized and reduced percentage decrease in FBA metrics, resembling the impact of training as in control cats, much more than the effect of retinal lesions in untrained animals. Possibly by virtue of visual training, ongoing anatomical reorganizations induced by retinal lesions are either halted or remodeled.

In MD patients, the best described long-term anatomical outcome of central retinal loss is a reduction in the volume of the gray matter in early visual cortical areas (e.g., [14, 24, 51, 59]) and of the LGN [30]. Since our MRI measurements of structural adaptations throughout the whole cat brain were acquired within the first 12 weeks after lesion induction, we did not find such significant changes in gray matter volume. An fMRI study on two MD patients by Baker et al. [10] revealed that parts of the visual cortex, including V1, that became unresponsive to central visual stimulation, can be activated by peripherally located visual stimuli, reflecting possible adjustments of the visual system to long-term central vision loss. We previously showed that exactly those cortical regions that are sensitive to motion and receive inputs from the peripheral retina retain a much stronger capacity to adapt to impairment of visual input during brain development [40, 41, 70]. In the present study, we showed that in adult cats, facilitation of perceptual motion-driven vision via full-field stimulation of the spared peripheral retina was evident 2 to 12 weeks after central retinal lesions. This superior visual performance of trained lesioned animals most likely relates to the observed partial preservation of white matter structure.

### The exceptional response to lesion and training—area V5/PMLS

Decisively, the only cortical area with FA values not affected by central retinal loss was the motion-sensitive area V5/PMLS. This observation is reminiscent of our previous findings in which area V5/PMLS also did not exhibit a decrease in neuronal activity based on the expression of the activity reporter gene *zif268*, even within its lesion projection zone, unlike other visual areas such as V1-V4, which showed an immediate and prolonged decrease in *zif268* expression in their lesion projection zone [15]. FBA analysis showed that the fiber density percentage decrease in area V5/PMLS up to 40% was equally strong in retinal-lesioned naive and retinal-lesioned trained cats. To better understand this result, one needs to remember that the FD metric relates to changes in the number of axons within a fiber bundle and to a microscopic estimate of the density of axons within a particular fiber population within the voxel [53, 65]. Therefore, an FD decrease can be interpreted as a reduction in the number of axons within the fiber bundles projecting to area V5/PMLS upon retinal lesion induction. Interestingly, no other FBA metric was changed, meaning that the fiber bundle diameter was unaffected. This fiber density reduction within area V5/PMLS may not result from axon degeneration, which would be reflected by a functionally altered fiber cross-section [54], but is related to fibers being recruited for other functions, as shown in blind subjects where V5 was activated by auditory stimuli [13, 57, 58]. Importantly, area V5/PMLS receives direct input via the pulvinar complex [47, 50, 55], leaving the response properties of V5/PMLS neurons relatively unchanged after lesions of the primary visual cortex [28, 63].

### Effects of visual training after central retinal lesions on white matter structure

#### Hippocampus

For all FBA metrics, we show a significant bilateral reorganization of white matter fibers reaching the hippocampus in all cats with retinal lesions. The role of the hippocampus in visual perception was demonstrated in patients with hippocampal lesions (e.g., [42, 56]). Patients showed impairment in scene discrimination tasks [42] and lack of memory for familiar pictures [56]. A decrease in FBA metrics was observed in the hippocampus of all lesioned cats, probably related to the limited exploration of the environment after such lesions. However, in retinal-lesioned trained animals, this percentage decrease was spatially limited to the FC and FDC metrics compared to retinal-lesioned naive cats. This finding might be the result of reinforced exploration of the visual scene by training. Although part of the familiar scene was taken away in retinal-lesioned trained and retinal-lesioned naive cats, the prelesion memory of eye movements [42] might have been preserved and strengthened by training, presumably halting the reduction of fiber density in retinal-lesioned trained cats.

#### Caudate nucleus

We detected lesion- and training-related changes in the caudate nucleus, the part of the striatum that is strongly innervated by dopaminergic neurons [46] and engaged in motivational control of saccadic eye movements [1, 12, 34, 55]. The fiber cross-section metric had a smaller percentage decrease, which was also spatially limited in retinal-lesioned trained cats compared to retinal-lesioned naive cats, most likely as a result of our training paradigm, using strong motivation by food reward to enhance visual exploration and discrimination. Supposably, training and learning utilize similar mechanisms, and human neuroimaging studies point to possible roles of the caudate nucleus in different forms of learning related to our study, including trial and error [18], visual classification [7] and category [61].

### General temporal pattern of white matter FA results

Training had an effect on lesion-induced fluctuations in FA values, as shown by decreased levels of FA in retinal lesion-trained compared to retinal lesion-naive cats. Similar sinusoidal temporal dynamics of FA values were previously described in training paradigms, with an initial increase, returning to baseline and even decreasing levels at the end of training (braille reading: [44],juggling: [60]). Interestingly, considering the swiftness of the increase in FA values and decreases in fiber density in the right hippocampus, the right hemisphere seems to react faster to the induction of retinal lesions, at least in the retinal-lesioned naive group. This unilateral, right response to lesions in naive cats appears consistent with the general understanding of a right hemisphere dominance in attention deployment [67]. Behavioral training may counteract this imbalance by stimulating global visual attention.

### White matter changes after retinal lesion induction in the dLGN and areas V1- 4

Only in the retinal-lesioned naive group the white matter fibers inside the optical tract and those reaching the dLGN were profoundly affected, as shown by temporal dynamics of FA levels and the fiber cross-section percentage decrease in the dLGN. This metric defines the macroscopic change in the cross-sectional area, perpendicular to the fiber bundle [54], and most likely reflects the anterograde and terminal degeneration of retinogeniculate afferents in the dLGN after lesion induction. The strongest increases in FA values in the dLGN were detected 2 weeks after lesion induction, returning to baseline at 8 weeks post lesion. Baekelandt et al. [9] previously found evidence for synaptic remodeling in the cat dLGN after central retinal lesioning. The timing of fluctuations observed in immunoreactivity for the synaptic density marker synapsin resembles our findings, with a peak at 3 weeks and a return to baseline levels by 7 weeks. A parallel increase in immunoreactivity for the marker of new synapse formation GAP-43 from 3 weeks onward substantiated the occurrence of rewiring in the dLGN even further. In line, cell counts did not reveal any neuronal loss in the lesion projection zone of the dLGN after such lesions [21]. The changes in synaptic connectivity most likely lead to dLGN receptive field displacements starting after 4 weeks and lasting for as long as 50 weeks, as shown electrophysiologically in retinal-lesioned cats [19, 20]. Interestingly, the temporal dynamics of growth-associated protein levels in the dLGN of the capuchin monkey MD model also closely reflect our FA dynamics in the dLGN, with an initial increase and return to baseline levels at 4 weeks postlesion survival (GAP43, GFAP and calcium-binding proteins [49]).

At the cortical level, the lesion projection zone in central V1-2 showed FA increases from 2 to 8 weeks, reaching baseline again by 12 weeks post lesion, as previously demonstrated neurophysiologically and neurochemically in V1 of retinal-lesioned cats [3, 25, 32]. Our FA results within V2-4 revealed that for retinal-lesioned naive animals, FA levels increased from 4 weeks postlesion and returned to baseline 8 weeks postlesion. Similar time-dependent dynamic *zif268* expression patterns were described for V2/18, V3/19, and V4/21a [15], with an initial decrease immediately after the lesions, followed by partial recovery over time.

## Conclusions

In conclusion, we demonstrate in our MD animal model that in the 1st weeks and months after central vision loss, visual capabilities benefit from applying a specific visual training approach and how this is reflected in a reduction in lesion-induced changes in specific white matter structures. Whether this finding is transferable to human patients needs to be considered in terms of intrinsic networks and behavior. Obviously, in humans, such tightly controlled retinal deficits do not occur and from experience we know how difficult it is to have visual deficit patients engage in behavioral testing, especially in a longitudinal study. Obviously, the cat’s visual system shows circuit similarities but also differences to humans [48]. Still, the comparison of results from different species is attractive to make good predictions regarding mechanisms involved in visual deficits to hopefully enable continuative human research and the design of new treatments. We propose that early behavioral therapy consisting of motion acuity training aimed at retinal peripheries may help to take over functions normally dependent on the central retina, such as fine detail analysis, in MD patients. Future in-depth tracing of molecular, cellular and circuit processes following retinal lesions in animal models and subsequent visual training, also in humans, might create new opportunities to rescue lost visual functions and unclose further insights into the neuronal mechanisms involved.

## Methods

### Animals and procedure

The first control MRI scannings were performed when the animals were 8 months old, at time point 0 (TP0, Fig. 1A). Then, all trained cats (Control Trained (CT, n = 5) and Retinal Lesioned Trained cats (RLT, n = 4) were familiarized with the automatic training apparatus for 3 weeks, as described in Zapaśnik and Burnat (2013), and pretrained. At the same time, retinal lesioned naive (RLN) cats were handled in the animal facilities. Following the induction of retinal lesions, next MRI scanning was performed after a 2-week recovery period (TP2, Fig. 1A). This time point was chosen as the first experimental condition because previous investigations have shown a clear functional [25] and maximal molecular response to the retinal lesion 2 weeks after lesioning [3]. After TP2, visual training began for the control trained and retinal-lesioned trained cats. Next MRI scannings were performed 4 weeks (TP4), 8 weeks (TP8) and 12 weeks (TP12) after lesion induction. During the whole training period, the control trained and retinal-lesioned trained groups were trained 5 days a week. Retinal-lesioned naive cats were also scanned at all these time points.

### Retinal lesion induction

Homonymous central retinal lesions were induced by photocoagulation (LOG-2 Xenon light photocoagulator, Clinitex) under ketamine/xylazine anesthesia (0.5 ml Ketalar, 0.2 ml Rompun, i.m.). Nictitating membranes were retracted with phenylephrine hydrochloride (5%), and pupils were widened with atropine sulfate (1%). Circular homonymous lesions with sharp borders and a 10° diameter were centered over the area centralis, as verified by fundus photography taken during lesion induction. After verification, in two cats the lesions were elongated for 2 degrees towards periphery by immediate second photocoagulation to adjust for perfect binocular overlap. There was no retinal bleeding observed in any of lesioned cats. This type of lesioning destroys all retinal cell layers, including the retinal ganglion cells, leading to the formation of a glial scar with sharp borders toward the surrounding normal retina [15, 20, 22]. Starting from the first postlesion day, the retinal-lesioned cats were observed behaviorally.

### Behavioral procedure and stimuli

For control trained and retinal lesioned trained cats, the motion discrimination training consisted of 17 tasks with increasing perceptual difficulty, visualized in the order of training in Table 1. Cats received food as a reward during training sessions, with *ad libitum* access to water in their home cages. Body weight was monitored every day before and after training to ensure a healthy condition and was kept at 90% of free-feeding body weight. The cats were trained in the two-choice apparatus with the food reward designed after Berkley [16]. For testing, the positive (S+, circle) and negative (S-, ellipse) stimuli were presented simultaneously. The animal, enclosed in the box, could see either the positive or negative stimulus (the left/right position was randomized) through one of the two translucent response keys 8.7 cm wide and 25 cm high. An occluder between the response keys prevented the animal from seeing both stimuli at once. The viewing distance was 21.5 cm, and both stimuli were placed in the center of the response keys. Pressing the response key for S+ provided a semiliquid reward made of mixed canned and dry animal food. Pressing the S- response key prolonged the time interval preceding the next stimulus presentation from 0.5 to 1 s. No correction was allowed. The difficulty of the tasks increased gradually, as depicted in Table 1. Pretraining consisted of familiarizing animals with the training procedure. The first pretraining task consisted of a circle built from white random dot kinematograms (RDK), with dots moving coherently downward at speed 40 deg/s (S+), and a circle with gray, stationary random dots (S-), both on a black uniform background. In subsequent tasks (1-17), S+ was a circle and S- an ellipse, displayed on the background, which was either uniform, with no RDK displayed (tasks 1 and 2) or built from random dots: stationary (task 3) or moving dots (tasks 4–16). Stimuli 1–2 and 8–16 were presented twice: in positive (white dots on black background) and negative (black dots on white background) contrast, as presented in Table 1. A two alternative, forced-choice procedure was used in all tasks. In tasks 1–7 and 10–14, daily sessions consisted of five 20 trial blocks for constant stimuli presentation, and the percentage of correct responses was calculated. The staircase method, where the difficulty of the task was automatically adjusted to the cat performance, was used to measure the thresholds: for the difference between background velocity and S+/- in tasks 8–9; for the difference between background coherence and S+/- in tasks 13–14, and for adjusting the S- ellipse aspect ratio to the S+ circle), showing the difference in shape between S+/- in tasks 15-17. Based on previously described superior performance of retinal-lesioned cats [15], each task was presented only for 2 consecutive days, then, irrespective of the animal’s performance, the new task was introduced in the order shown in Table 1.

### Anesthesia and MRI data acquisition

Animals were premedicated i.v. and anesthetized with ketamine (3–6 mg/kg), butorphanol (0,1–0,5 mg/kg) and dexmedetomidine (0,03–0,05 mg/kg) in the animal facilities to allow safe transport to the MRI scanner, where isoflurane was administered via a mask. During the neuroimaging protocol, physiological parameters (breathing rate and temperature) and the general condition of animals were monitored. To maintain the body temperature within physiological ranges, animals were placed on a heating pad operated by a warm water control system. Head fixation foam pads were used to minimize artifacts related to motion. All MRI data were collected using a 7T horizontal MR system (Bruker MRI BioSpec 70/30) equipped with a custom-made, single-channel loop coil (diameter = 55 mm) placed directly on the cat's head by removable tape. The coil was attached to a BRUKER preamplifier, which is a part of the “Flexible Surface Coils with dedicated Preamplifier” setup. Our coil had a detuning capability to prevent it from resonating with the RAPID coil during the transmission phase. The loop-coil receiver in conjunction with the RAPID transmitter proved to be the best setup with regard to signal-to-noise ratio (SNR) in a cat’s brain. First, the localizer scannings (TriPilot) were acquired to access the cats’ position and place them in the scanner appropriately. Then, a B0 field map was acquired to measure static field inhomogeneities, after which shimming was performed for correction. Next, TurboRare T2-weighted structural scannings were collected. Eventually, DW images with 81 uniformly distributed diffusion gradient directions (b = 500 s/mm2) and 3 non-DW (b = 0 s/mm2) datasets were acquired twice, first in the AP phase encoding direction, followed by the reversed-phase encoding direction (PA). The DWI scanning parameters were as follows: FOV 80x80 mm, echo time (TE) 35 ms, repetition time (TR) 10000 ms, acquisition matrix size 128 × 128, b-value = 500 s/mm2, and voxel size: 0.625 × 0.625 × 0.8 mm. Each imaging session was approximately 1 h 30 min.

### Volumetric analysis

Each scan was processed with the FMRIB Software Library (FSL, Analysis Group, Oxford—[39]) to extract the brain from the skull (bet2), followed by manual detection of errors in skull stripping. Next, the Serial Longitudinal Registration (SLR—[5]) using Statistical Parametric Mapping 12 (SPM12, The Wellcome Centre for Human Neuroimaging, Institute of Neurology, London) was performed to create an average “within subject” template for each animal,besides the average midpoint, the SLR generates: deformation fields (y), jacobian determinant (j) and divergence of velocity (dv) maps for within-subject deformations. Next, the averaged template for each cat (from SLR) was fed into the “build template parallel” function of the Advanced Normalization Tools (ANTs—[8]) to obtain a “between subject” population-based 3D volume used for segmentation in SPM12; this segmentation step segments each cat’s average image to obtain a gray, white and corticospinal fluid (CSF) mask (c1, c2, c3) and, in parallel it generates the same tissue masks resliced and prepared to generate a DARTEL template (rc1, rc2, rc3) that considers individual brain’s deformation (in SPM12—[4]). Eventually we decided to weight the jacobian determinant (obtained as an output from SLR) from each animal with the c1 mask (generated during segmentation) in subject space to obtain data presenting volumetric changes within gray matter. As a conclusion for preprocessing, we warped each subject space data to a common space (i.e. DARTEL template from previous steps).

### Voxel based morphometry—longitudinal group analysis.

To identify and compare areas of different gray matter concentration [6] we performed a flexible factorial analysis [27] including all MRI measurements time points (TPs) and all groups of animals. Time has been added as the first factor (5 levels: TP0, TP2, TP4, TP8, TP12) and the group as the second factor (2 levels: trained and untrained). Each TP was matched with the others within and between the two groups. An explicit mask was set to exclude frontal cortex and cerebellum from the tests. Mask was created manually using ITK-Snap [69]. Family wise error corrected p-values were used to account for multiple comparisons (p <0.05, FWE). No significant differences in VBM analysis were detected.

### DWI data preprocessing- FBA analysis

Before the implementation of the fixel based analysis, data was converted from Bruker ParaVision 5.1 image matrices to NIFTI using an inhouse MATLAB script. Additionally, PA encoded acquisition was mirrored to match anatomically AP with SPM build-in functions. Further preprocessing steps, typical for FBA were performed using MRtrix3 (https://www.mrtrix.org/): conversion to mif. format (mrconvert), diffusion gradient table check (*dwigradcheck*), denoising (*dwidenoise*), Gibbs ringing correction (*mrdegibbs*), motion and distortion correction (*dwipreproc*). DWI-based masks were created (*dwi2mask*) for eachsubject, followed by bias field correction (*dwibiascorrect*) and image normalization (*dwiintensitynorm*). Fractional Anisotropy maps (FA) were calculated with the *dwiintensitynorm* script, which also performs registration to a group-wise template and creates a white matter mask. Next, computation of the average white matter response function was performed (*dwi2response*), followed by group average response function estimation (*average_response*). After this step, data was upsampled by a factor of 2 (*mrresize*). Then, Fiber Orientation Distribution (FOD) estimation was performed individually (*dwiextract*) using multi tissue constrained spherical deconvolution. Subsequently, a study specific template was created by two-step spatial normalization: first intra- and then inter-subject template generation was performed accordingly. Further, a template mask was created, which is the smallest possible mask out of all data, and all individual masks were normalized to the template mask. Then, a white matter template analysis fixel mask was computed. In this step, fixels from the FOD template were segmented. As the result, the fixel mask that defines the fixels for which statistical analysis will later on be performed was defined. Eventually, fixel specific measures of fiber density (FD) and fiber bundle cross-section (FC) as well as a combined measure (FDC, multiplication of FD and FC) were derived.

### Statistical analysis

#### Fixel-Based Analysis

Fixel-based smoothing and statistical inference were performed using connectivity-based fixel enhancement (CFE). Familywise error (FWE)-corrected p values were then assigned to each fixel, testing over 5000 permutations, using nonparametric permutation testing, and familywise error correction for multiple-comparison corrections; values of *p* < 0.05 were considered statistically significant. Significant fixels were then visualized with the mrview tool in Mrtrix3 on the population template-based tractogram (Figure 1B), in which streamlines were cropped to only significant fixels (Figure 1C), to better visualize the regions implicated in changes. Streamlines were colored by the effect size expressed as a significant percentage decrease relative to the control group (Figure 1D). Regions with significant percentage decreases are shown in Figure 2A. All significant differences revealed with FBA analysis were obtained by grouping time points together for each group of cats separately and then by comparing results between groups. Importantly, no differences between groups of cats were revealed at baseline measurement at TP0.

#### Fractional Anisotropy

Individual FA maps were analyzed with SPM software, where comparisons of the mean FA values of the extracted ROIs were performed. A full-factorial GLM analysis was performed with Bonferroni correction, with hemispheres (left and right), timepoints (TP0, TP2, TP4, TP8, TP12), ROIs (12) and groups of cats included as categorical factors in the model. The GLM analyses were performed using Statistica software (1995–2020 TIBCO Software, Inc.).

All DWI results, FBA and FA, were brought to common space as ROIs for FA and FBA were drawn and presented on the same population template. Selected slices of this population template are shown in Figure 1B and C. Based on our previous experimental data performed on the same cat MD model [15] and results of current FBA analysis we assessed five visual cortical areas and the dLGN [66]. ROIs were manually delineated in FSLeyes software based on Burnat et al. [15] for V1-2 central and peripheral (17,18), V2-3 (18,19), V4 (20ab), and V5/PMLS, as shown in Figure 2B.

#### Behavioral data

To compare the behavioral performance of the control trained and retinal lesioned trained cats, a one-way non-parametric analysis of variance ANOVA on ranks (Kruskal-Wallis test) was used. Significant differences between groups in tasks and within groups between tasks are indicated by: *p ≤ 0.05, **p ≤ 0.01 and ***p ≤ 0.001, Dunn’s Multiple Comparison post-hoc test was performed.

## Data Availability

The datasets generated and analyzed during the current study are publicly available and are available from the corresponding author upon reasonable request.

## Abbreviations

7 T MRI: 7 Tesla magnetic resonance imaging
CT: Control trained cats
dLGN: Dorsal lateral geniculate nucleus
DTI: Diffusion tensor imaging
FA: Fractional anisotropy
FBA: Fixel based analysis
FC: Fiber cross-section
FD: Fiber density
FDC: Fiber density and cross section
fMRI: Functional magnetic resonance
FWE: Familly wise error
MD: Macular degeneration
MRI: Magnetic resonance imaging
RDK: Random dot kinematogram
RLN: Retinal-lesioned naive cats
RLT: Retinal-lesioned trained cats
ROI: Region of interest
S-: Negative stimulus
S +: Positive stimulus
TP: Time point
V1: Primary visual cortex
V5/PMLS: Motion sensitive visual area/posteromedial lateral suprasylvian cortex
